# Editorial Comments to Impact of Adjuvant Radiotherapy and Mitotane on Survival in Localized Adrenocortical Carcinoma: A Retrospective Cohort Study

**DOI:** 10.1111/iju.70355

**Published:** 2026-01-19

**Authors:** Kento Morozumi

**Affiliations:** ^1^ Department of Urology Tohoku Medical and Pharmaceutical University Miyagi Japan

Adrenocortical carcinoma (ACC) is an uncommon and highly aggressive malignancy for which evidence‐based postoperative management strategies remain limited [1]. Local recurrence after surgery is frequent, and although mitotane‐based systemic therapy forms the foundation of current practice, the optimal approach to achieving durable locoregional control continues to evolve [2]. In rare cancers such as ACC, where prospective randomized trials are difficult to conduct, even incremental clinical evidence derived from retrospective or multi‐institutional experiences plays an important role in shaping contemporary management strategies.

This study provides valuable insight into the role of adjuvant radiotherapy (RT) following curative‐intent surgery. The reported improvement in local control is clinically meaningful and supports RT as a rational tool for managing microscopic residual disease [3]. At the same time, the absence of a corresponding improvement in disease‐free or overall survival highlights a central feature of ACC: distant metastasis remains the dominant mode of treatment failure [4]. Thus, RT appears effective within the local field, yet insufficient on its own to alter systemic disease trajectory.

From a clinical standpoint, the presented data support selective use of adjuvant RT, particularly in patients with pathological features such as capsular invasion, vascular infiltration, or positive or close surgical margins. These findings help position RT as one component within a broader multimodal strategy and suggest that greater therapeutic gains may be achieved when RT is integrated with systemic approaches. The development of more effective pharmacologic agents, as well as advances in radiation delivery technique, represent important pathways for future investigation [5].

Despite the retrospective design and the inherent challenge of limited patient numbers, this study provides important context for decision‐making in postoperative ACC management. It clarifies situations in which adjuvant RT can provide meaningful benefit and offers a practical reference for clinicians facing real‐world therapeutic uncertainty. Ultimately, this work reinforces the role of RT as a contributor to locoregional control and a foundation upon which future combined‐modality treatment strategies may be constructed.

## Author Contributions

**Kento Morozumi:** contributed to the conceptualization of the study and the writing of the manuscript.

## Conflicts of Interest

The author declares no conflicts of interest.

## Linked Articles

Impact of Adjuvant Radiotherapy and Mitotane on Survival in Localized Adrenocortical Carcinoma: A Retrospective Cohort Study. https://doi.org/10.1111/iju.70319.
